# Migration-Related Characteristics and Children’s Consumption of Ultra-Processed Foods

**DOI:** 10.3390/foods15040765

**Published:** 2026-02-19

**Authors:** Josep A. Tur, Aristides Machado-Rodrigues, Daniela Rodrigues

**Affiliations:** 1 Research Group on Community Nutrition & Oxidative Stress, University of the Balearic Islands—IUNICS, 07122 Palma de Mallorca, Spain; pep.tur@uib.es; 2 CIBEROBN (Physiopathology of Obesity and Nutrition), Instituto de Salud Carlos III, 28029 Madrid, Spain; 3 Health Research Institute of Balearic Islands (IdISBa), 07120 Palma de Mallorca, Spain; 4 Research Centre for Anthropology and Health (CIAS), University of Coimbra, 3001-401 Coimbra, Portugal; a.machado-rodrigues@fcdef.uc.pt; 5 Faculty of Sport Sciences and Physical Education, University of Coimbra, 3040-248 Coimbra, Portugal; 6 Interdisciplinary Centre for the Study of Human Performance (CIPER), University of Coimbra, 3040-248 Coimbra, Portugal; 7 Department of Life Sciences, University of Coimbra, 3000-456 Coimbra, Portugal

**Keywords:** ultra-processed foods, children, migration, Portugal

## Abstract

Ultra-processed foods (UPFs) have become a dominant component of contemporary food environments worldwide. Their consumption is socially patterned, with higher intakes frequently observed among children from socioeconomically disadvantage families, highlighting a critical dimension of dietary inequality. International migration is another major social determinant of familial diet; however, few studies have examined how migration-related characteristics is associated with children’s UPF consumption in Portugal. This study assessed the association between migration-related characteristics, namely parental nationality and whether the child had always lived in Portugal, and UPF consumption among young children. Cross-sectional analysis of data from the prospective ScreenHealth cohort (5.6 ± 1.0-year-old children; *n* = 682; 52.1% male) included information on migration status, dietary intake, and covariates (age, parental education). Children with two foreign parents or who had not always lived in Portugal showed higher odds of consuming several UPF items. These associations were only partially attenuated after adjustment for socioeconomic indicators like parental education. Findings are descriptive and should be interpreted with caution, particularly given the small sample sizes of some immigrant subgroups and the cross-sectional design, but they provide novel insights into early-life dietary patterns and highlight population groups that may be differentially exposed to UPF-rich food environments during early childhood.

## 1. Introduction

Ultra-processed foods (UPFs) have become a dominant component of contemporary food environments worldwide [1]. Supermarkets, digital media, and advertising spaces are increasingly saturated with packaged snacks, sugar-sweetened beverages, ready-to-eat meals, and industrial baked goods. Reflecting this ubiquity, the contribution of UPFs to total dietary intake has risen substantially over recent decades, particularly among children and adolescents. According to UNICEF’s Child Nutrition Report 2025 [2], around 60% of adolescents globally and more than half of children aged 6–23 months in several low- and middle-income countries consumed a sweet food or beverage the previous day. In many high-income countries, UPFs now account for at least half of total daily energy intake among adolescents [2].

Under the NOVA classification system, UPFs are defined as industrial formulations composed primarily of refined ingredients—such as sugars, fats, and protein isolates—combined with additives that enhance palatability, appearance, and shelf life. These products are designed to be hyper-palatable, convenient, and aggressively marketed, often displacing fresh or minimally processed foods and traditional dietary practices. A growing body of evidence links high UPF consumption to adverse health outcomes, including excessive energy intake, poor diet quality, and exposure to potentially harmful additives and food-contact chemicals [3]. While much of the literature focuses on adults, emerging evidence in children associates UPF intake with increased body weight, adiposity, unfavorable lipid profiles, and susceptibility to mental health disorders [4,5,6,7,8,9]. Moreover, it seems that children are uniquely vulnerable to the impacts of UPFs because their bodies and brains are developing and they are more sensitive to the nutritional deficiencies and metabolic disruptions caused by UPFs [10]. Early exposure to UPFs may also reinforce dietary patterns that persist in adulthood, increasing the risk of chronic disease across the life course [9,11].

Early childhood represents a particularly vulnerable period for the establishment of dietary preferences and long-term eating habits. Recent estimates indicate that UPFs contribute approximately 47% of daily energy intake among toddlers and nearly 60% among school-aged children [9]. Importantly, UPF consumption is socially patterned, with higher intakes frequently observed among children of families facing socioeconomic disadvantage, highlighting a critical dimension of dietary inequality [9,12,13].

International migration is another major social determinant of diet and nutrition. Globally, more than 281 million people live outside their country of birth, and migration is often accompanied by profound changes in food environments, social norms, and daily routines [14,15,16]. Data from the EU27 countries show that, between 2012 and 2021, Portugal was one of the countries where the number of immigrants increased most compared to the resident population: only Malta, Estonia and Lithuania had greater growth in this indicator. Recently, in Portugal, data from the Fundação Francisco Manuel dos Santos [17] revealed there are about 11% of immigrants which is twice as much as ten years ago. The most representative nations are Brazil (29.3%), UK (6%), Republic of Cabo Verde (4.9%); Italy (4.4%), India (4.3%), Romania (4.1%), and Ukraine (3.9%).

Numerous studies have documented dietary shifts following migration, with migrants frequently moving away from traditional diets rich in minimally processed foods toward patterns characterized by greater consumption of processed and UPF products [15,18]. Processes such as dietary acculturation, duration of residence, household composition, and exposure to the host country food system have all been shown to influence food choices, though findings are heterogeneous and context dependent [19]. Because young children’s diets are largely shaped by parental food choices, household food availability, and caregiving practices, migration-related dietary changes observed among adults are likely to directly influence children’s eating behaviors. In line with this, parental migration status, more than parental education, was a predictor of the intake of unhealthy food items including sweets, crisps, cakes, ice creams, soft drinks, juice, and fast food by 6-year-old children in Sweden [12].

In population-based studies, these processes are often examined indirectly using migration-related indicators such as parental country of origin or residence history, which function as proxy measures rather than direct assessments of acculturation or cultural change [20]. Accordingly, associations observed using such indicators should be interpreted as reflecting intersecting dimensions of migration experience, including structural constraints, household food practices, and exposure to the host food environment, rather than a single underlying mechanism [21].

Despite growing interest in migration and diet, evidence focusing specifically on young children remains limited, particularly in European settings. Portugal has experienced a steady increase in its migrant population in recent decades [22], yet few studies have examined how migration-related characteristics shape children’s consumption of UPFs. Therefore, the aim of this study was to assess the association between migration-related characteristics, namely parental nationality and whether the child had always lived in Portugal, and the consumption of UPF items among young children, independent of socioeconomic status (SES) as measured by parental education. We hypothesized that migration-related characteristics would be positive and significantly associated with consumption of several UPF items among Portuguese young children. Understanding how migration intersects with early dietary behaviors may help to identify vulnerable groups and inform culturally sensitive nutrition policies and interventions.

## 2. Methods

### 2.1. Study Design and Sample

A cross-sectional analysis of data collected at the second-year follow-up visit of the prospective ScreenHealth cohort (https://screenhealth.uc.pt/) was conducted. The ScreenHealth Project is the first longitudinal study in Portugal to investigate screen media use trajectories in young children, their interactions with other behaviors (e.g., physical activity, sleep, and dietary patterns), and their impact on childhood obesity and mental health.

**Figure 1:**
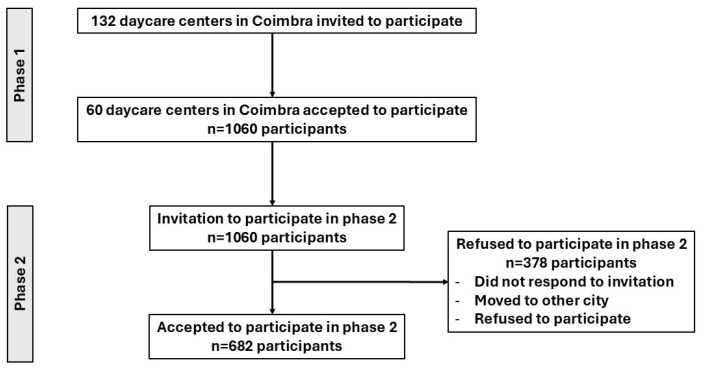
Flowchart of the study.

In 2024, parents of children attending 132 daycare centers and preschools in Coimbra were invited to participate in the project. A total of 60 establishments—both private and public—and 1050 child–parent dyads were included in the first wave (April–June 2024). All 1050 participants enrolled in phase 1 (baseline) were invited to participate in phase 2, which included 682 children. Dropouts included participants who did not respond to the phase 2 invitation, moved to another city or country, or refused to participate. Phase 2 data collection took place between January and June 2025. A flowchart showing this process is presented in Figure 1.

All parents provided written informed consent. The study protocol was approved by the Ethics Committee of the Interdisciplinary Research Institute of the University of Coimbra (CEIIIUC; REF 1_ID312, 26 July 2023) and was conducted under the supervision of the University’s Data Protection Officer.

All variables were collected using questionnaires completed by parents at home. Parents were asked to return the complete questionnaires to the schools in a sealed envelope to ensure data anonymity.

### 2.2. Migration Status—Independent Variables

Parents were asked to report whether their child had “always lived in Portugal” (no/yes). They also reported their country of origin, which was used to create three variables: (1) Parents’ nationality, classified as two foreign parents, one foreign parent, or two Portuguese parents (no foreign); (2) mother’s origin; and (3) father’s origin.

Four categories were defined for the mother’s origin: Africa (South Africa, Angola, Cape Verde, Nigeria, and São Tomé), South America (Argentina, Brazil, Colombia, and Venezuela), Europe (France, Italy, Luxemburg, Poland, Russia, United Kingdom, Sweden, and Ukraine), and Portugal. The United States of America was excluded due to small representation (*n* = 1). For fathers, the same categories were derived: Africa (Angola, Cape Verde, Côte d’Ivoire, Guinea, Mozambique, Nigeria, and São Tomé), South America (Argentina, Colombia, and Venezuela), Europe (France, the Netherlands, Italy, Luxemburg, and Moldova), and Portugal. The United States of America (*n* = 1), Canada (*n* = 2), and China (*n* = 1) were excluded due to small sample sizes.

### 2.3. Dietary Intake and UPF Consumption—Dependent Variables

UPF consumption was assessed using the NOVA-UPF screener, a brief, food-based questionnaire originally developed and validated in Brazil to rapidly assess exposure to UPFs based on consumption the previous day [23]. This tool consists of a predefined list of UPF subgroups, with respondents indicating whether any item from each subgroup was consumed on the day before (yes/no) and is designed to capture frequency of exposure rather than quantities or energy intake. A Portuguese version of the NOVA-UPF screener has been developed and validated for the Portuguese population based on national dietary intake data and a structured expert consensus process to ensure cultural relevance and content validity [24]. This validation demonstrated good agreement between the screener score and the dietary contribution of UPFs, supporting its use as a rapid assessment tool. This version has previously been applied by our research team in a sample of adolescents aged 12–17 years [25]. In the present study, a list of 27 predefined UPFs items, adapted for use in children and reported by parents, was used. Items were grouped into three categories: beverages (items 1–6), products that replace or accompany meals (items 7–17), and products often consumed as snacks (items 18–27). The complete list of items is presented in Table 1. This tool is simple and quick to complete and requires a low respondent burden, making it feasible for rapidly quantifying UPFs consumption.

### 2.4. Covariates

Additional information was obtained: age (in years) and educational level of the mother and the father (below university, university degree) as a proxy for SES; however, the latter of these captures only one dimension of socioeconomic circumstances and may not fully reflect income, employment stability, or material resources, particularly among migrant families.

### 2.5. Statistical Analysis

Descriptive statistics were used to characterize the sample. Continuous variables were summarized using means and standard deviations (SD), while categorical variables were presented as frequencies and percentages. Bivariate associations between children’s consumption of UPFs and maternal origin were explored using chi-square tests of independence. Fisher’s exact test was applied when expected cell counts were below 5. These analyses were conducted for descriptive and exploratory purposes, given the small sample size in some origin groups.

Multiple logistic regression models were used to examine the associations between children’s consumption of UPFs (no/yes, consumption on the previous day) and migration-related exposure variables, namely whether the child had always lived in Portugal and parental nationality. Three sequential models were estimated: Model 1 (unadjusted); Model 2, adjusted for child sex and age; and Model 3, further adjusted for SES, operationalized by maternal and paternal educational level. Odds ratios (OR) and 95% confidence intervals (95% CI) were reported. All analyses were performed using IBM SPSS Statistics version 28.0, and statistical significance was set at *p* < 0.05.

## 3. Results

This study uses data collected in the second wave (phase 2; 2025), including 682 children (mean age: 5.6 ± 1.0 years), of whom 355 (52.1%) were boys (Table 2). Most children had two Portuguese parents (83.2%), followed by children with one Portuguese and one foreign parent (8.7%) and those with two foreign parents (8.1%). Although 112 children had at least one non-Portuguese parent, only 55 (8.1%) had not always lived in Portugal. Foreign mothers were predominantly of South American origin (6.7%), followed by African (3.1%) and other European origins (2.7%). Similar distributions were observed for fathers, with 6.6% of South American origin, followed by African (3.3%) and other European origins (1.8%).

**Figure 2:**
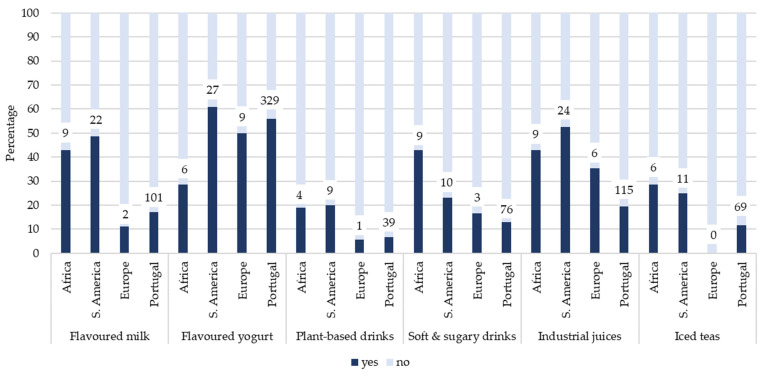
Prevalence (%) of beverage-related ultra-processed consumption on the previous day among children, stratified by mother’s origin. Numbers above bars represent the number of children consuming each item on the previous day. Total sample sizes were Africa (*n* = 21), South America (*n* = 45), Europe (*n* = 18), and Portugal (*n* = 588).

**Figure 3:**
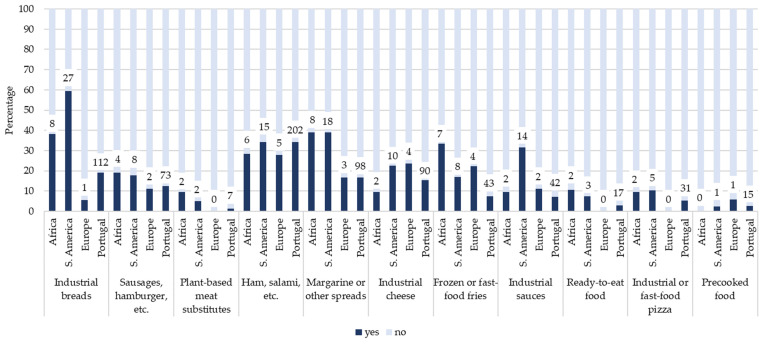
Prevalence (%) of ultra-processed food products that replace or accompany meals on the previous day among children, stratified by mother’s origin. Numbers above bars represent the number of children consuming each item on the previous day. Total sample sizes were Africa (*n* = 21), South America (*n* = 45), Europe (*n* = 18), and Portugal (*n* = 588).

**Figure 4:**
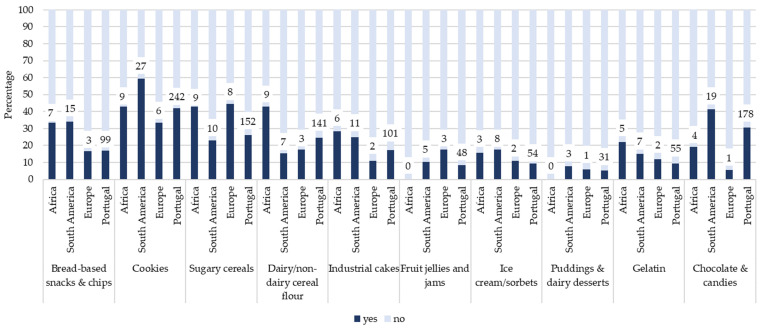
Prevalence (%) of ultra-processed food products often consumed as snacks on the previous day among children, stratified by mother’s origin. Numbers above bars represent the number of children consuming each item on the previous day. Total sample sizes were Africa (*n* = 21), South America (*n* = 45), Europe (*n* = 18), and Portugal (*n* = 588).

Figure 2, Figure 3 and Figure 4 illustrate the prevalence of UPF consumption on the previous day according to mother’s origin, grouped into beverages (Figure 2), and products that replace or accompany meals (Figure 3), and products often consumed as snacks (Figure 4). Overall, children with mothers of African and South American origin tended to report higher consumption of several UPF items across all three categories compared with children of Portuguese origin. Differences were particularly evident for beverages and meal-related products.

Regarding beverages (Figure 2), flavored milk (*ꭓ*^2^ = 39.19, *p* < 0.001) and industrial juices (*ꭓ*^2^ = 58.42, *p* < 0.001) were more frequently consumed among children with African and South American parental origin, whereas consumption was consistently lower among children of Portuguese origin. Soft and sugary drinks, iced teas and plant-based drinks showed lower overall prevalence but followed similar patterns.

In the category of products that replace or accompany meals (Figure 3), marked differences were observed for industrial breads (*ꭓ*^2^ = 52.51, *p* < 0.001), margarine or other spreads (*ꭓ*^2^ = 34.50, *p* < 0.05), and frozen or fast-food fries (*ꭓ*^2^ = 49.64, *p* < 0.01), with higher consumption among children with African and South American mothers’ origin compared with Portuguese-origin children. Consumption of plant-based meat substitutes, ready-to-eat foods, and industrial sauces was low overall but relatively higher among children of non-Portuguese origin.

For products often consumed as snacks (Figure 4), higher proportions of consumption among children with African and South American mother origin were observed for bread-based snacks (*ꭓ*^2^ = 32.48, *p* < 0.05), sugary cereals (*ꭓ*^2^ = 28.79, *p* < 0.05), and gelatin (*ꭓ*^2^ = 38.32, *p* < 0.05), while children of Portuguese origin generally reported lower consumption across these items. Similar results were found for father’s origin.

Table 3 shows the associations between children’s consumption of UPF items and having not always lived in Portugal, with children who had always lived in Portugal as the reference group. While adjustment for parental education attenuated several associations, increases in effect estimates were observed for some outcomes in fully adjusted models. In crude analysis, children who had not always lived in Portugal had significantly higher odds of consuming several UPFs. These associations were largely unchanged after adjustment for sex and age (Model 2). After additional adjustment for parental education (Model 3), higher odds of consumption remained statistically significant for industrial juices, industrial breads, plant-based meat substitutes, industrial sauces, and industrial cakes. Associations with flavored milk, plant-based drinks, margarine or other spreads, frozen or fast-food fries, and cookies were attenuated and no longer statistically significant after the adjustment. Overall, not having always lived in Portugal was associated with a higher likelihood of consuming several UPFs, particularly staple and meal-related products, even after accounting for sociodemographic factors.

Table 4 shows the association between parental nationality and children’s consumption of selected ultra-processed food items, using children with two Portuguese parents as the reference group. In crude analysis, children with two foreign parents showed significantly higher odds of consuming several ultra-processed products. These associations largely persisted after adjustment for child sex and age (Model 2). After further adjustment for parental education (Model 3), several associations remained statistically significant among children with two foreign parents, including consumption of flavored milk, plant-based drinks, industrial juices, industrial breads, margarine or other spreads, frozen or fast-food fries, industrial sauces, and cookies. In contrast, associations with soft and sugary drinks, ready-to-eat foods, bread-based snacks, industrial cakes, and gelatin were attenuated and no longer statistically significant after the final adjustment.

Children with one foreign parent showed fewer significant associations. After full adjustment, higher odds of consumption remained only for industrial cheese and fruit jellies and jams, whereas associations observed in crude models for other items were no longer significant. Overall, children with two foreign parents consistently exhibited higher odds of consuming several UPF items compared with children of two Portuguese parents even after accounting for sociodemographic factors, whereas associations for children with one foreign parent were weaker and less consistent.

## 4. Discussion

This study examined how migration-related characteristics are associated with the consumption of UPFs among young children in Portugal. Overall, children with a migration background, particularly those with two foreign parents or who had not always lived in Portugal, showed a higher likelihood of consuming several UPF items. Adjustment for parental educational level attenuated several associations, indicating that SES partially explains differences in UPF consumption between migrant and non-migrant families. However, parental education captures only one dimension of socioeconomic circumstances and may not adequately reflect other structurally relevant factors, such as household income, employment stability, and neighborhood food environments. Therefore, the persistence of some associations after adjustment may not fully capture the range of structural and material constraints shaping dietary behaviors. Moreover, as the NOVA-UPF screener captures recent exposure rather than habitual intake, the observed migration-related differences may reflect systematic differences in day-to-day food environment and routines rather than stable long-term dietary patterns alone. Nevertheless, present findings are consistent with previous studies reporting differences in dietary habits between migrant and non-migrant populations in German and Sweden [26,27]. Similarly, Canadian children aged 6 to 12 years whose mothers were family-class immigrants were shown to consume more UPFs than their non-migrant counterparts [28]. Together, these findings suggest that children with a migration background may be differently exposed to less favorable aspects of Western food environments, including higher availability and consumption of industrial food products.

Higher UPF consumption among children from migrant families may reflect the combined influence of food environments, daily routines, and household food practices shaped by migration-related circumstances. Families facing new social and economic contexts following migration may have greater exposure to ultra-processed products that are widely available, affordable, shelf-stable, and heavily marketed in the host country food environment. These products often require minimal preparation and are readily accepted by children, making them practical choices in the context of time constraints and structural barriers faced by migrant households. Nevertheless, dietary behaviors among migrant populations are shaped by multiple factors, including degree of integration, duration of residence, generation status, and language proficiency [16,29,30,31].

Migrant populations are disproportionately affected by food insecurity (FI) [32], which is influenced by economic constraints, educational background, cultural factors, and changes in lifestyle following migration. In Portugal, immigrant populations, particularly those from non-European countries, show higher FI prevalence compared to the native population [33]. FI has been consistently associated with poorer diet quality and greater reliance on energy-dense, nutrient-poor foods among children, and may contribute to higher UPF exposure in socioeconomically disadvantaged households, including many migrant families [34]. Neighborhood food environments are also socially patterned and disproportionately affect migrant households [19]. Although these factors were not directly measured in the present study, they may contribute to the residual associations observed after adjustment for parental education.

The stronger and more consistent associations observed among children with two foreign parents suggest that household-level factors and shared dietary practices play an important role in shaping early dietary behaviors. When both parents share similar migration experiences and food-related constraints, these may be more consistently reflected in everyday meal choices and food availability within the household. This finding highlights the importance of considering parental composition, rather than migration status alone, when examining children’s diets.

Children who had not always lived in Portugal also showed higher odds of consuming several UPF items, particularly beverages and products commonly integrated into daily meals, such as industrial breads and sauces. This pattern may reflect differential exposure to commercially available and packaged foods during early periods of residence in the host country, when families are navigating new food systems and institutional contexts. Changes in children’s food exposure related to schooling, childcare settings, and household circumstances may contribute to shifts in dietary patterns. However, evidence from non-US-born adults suggests that UPF consumption may change with longer residence in the host country [35], indicating that the relationship between migration-related exposures and UPF intake may be complex and context dependent. Importantly, the migration-related indicators used in this study should be interpreted as composite proxies encompassing multiple intersecting dimensions of migration experience, rather than as measures of acculturation or cultural change per se.

Adjustment for parental educational level reduced the magnitude of several associations, indicating that it partially explains differences in UPF consumption between migrant and non-migrant families. Parental education may influence nutrition knowledge, food purchasing behaviors, and awareness of dietary recommendations. Additionally, residence in disadvantaged food environments, such as food deserts, may further limit access to healthier food options [36]. Previous studies have shown that lower parental education and income are often associated with higher UPF consumption among children [37,38]; however, evidence from Mediterranean and other high-income countries suggests that higher UPF intake may also occur among families with higher educational levels, particularly in younger children [39]. This variability underscores the complexity of socioeconomic gradients in UPF consumption and suggests that SES alone does not fully account for migration-related differences.

In some outcomes, associations between migration-related characteristics and UPF consumption became stronger after adjustment for parental education. This pattern may reflect negative confounding, whereby socioeconomic indicators such as education mask underlying structural disadvantages within migrant families, particularly in contexts where educational attainment does not translate into income, employment stability, or food access. However, these enhanced associations should be interpreted with caution and not taken as evidence of stronger effects, since they may also reflect the low prevalence of some outcomes.

Notably, migration-related differences were more pronounced for beverages and staple-like products, such as industrial breads, sauces, and spreads, than for discretionary snack foods. These items are typically consumed as part of routine meals and may therefore be more sensitive to household-level food practices and structural constraints. In contrast, the consumption of sweets and snack foods was more heterogeneous across groups, possibly reflecting widespread availability and consumption among children regardless of migration background. Given that UPF intake has increased substantially during early childhood, with toddlers and school-aged children obtaining approximately 47% and 59% of their daily energy intake from UPFs, respectively [12], examining UPF subcategories provides useful descriptive insights into patterns that may contribute to dietary inequalities.

## 5. Strengths and Limitations

This study has several strengths, including a relatively large sample of preschool-aged children, the use of a standardized tool to assess UPF consumption, and the consideration of multiple migration-related indicators. The use of sequential regression models allowed for a nuanced examination of potential confounding by sociodemographic factors. However, some limitations should be acknowledged. The cross-sectional design precludes causal inference, and dietary intake was assessed based on parental report of consumption on the previous day, which may not reflect usual intake. Although the NOVA-UPF screener has been validated for use in the Portuguese adult population, its application in preschool-aged children relies on parental report and has not been formally validated in this age group. In addition, the screener assesses consumption on the previous day using binary responses, which captures recent exposure to UPFs rather than habitual intake, portion size, or energy contribution. Given that young children’s eating behaviors may vary according to daily routines, childcare settings, and occasional events, this single-day measure may reflect short-term situational consumption as well as underlying dietary patterns. Additionally, some migration subgroups were small, limiting statistical power and requiring cautious interpretation of subgroup analyses. In these subgroups, percentage estimates may appear unstable, and visual differences should be interpreted in light of the underlying absolute counts. Residual confounding by unmeasured factors, such as household income or food insecurity, cannot be ruled out, and the study sample is not fully representative of all immigrant children living in Portugal. This study is subject to potential selection bias due to loss to follow-up between the baseline and second wave of the cohort. Given that migration-related factors such as residential mobility, housing instability, and language barriers are associated with study participation and retention, it is possible that families with a migration background were disproportionately lost to follow-up. However, migration-related characteristics were not collected at baseline, precluding formal assessment of differential attrition or application of attrition weighting strategies. Consequently, the observed associations may reflect the experiences of children from more stable or reachable migrant households, and the magnitude of migration-related differences in UPF consumption may be underestimated or otherwise biased.

Despite these limitations, the findings provide relevant insights for public health by identifying population groups that may be differentially exposed to UPF-rich food environments during early childhood. As dietary habits are established early in life, patterns warrant attention from a prevention perspective, particularly in settings such as daycare and preschool environments where structural interventions may help reduce disparities in food exposure. Future research should incorporate longitudinal designs with repeated dietary assessments to better distinguish short-term situational consumption from habitual dietary patterns. Collecting detailed information on household income, childcare arrangements, and language accessibility would allow for a more comprehensive assessment of the structural determinants of UPF consumption among migrant families. Mixed-methods approaches may also help contextualize quantitative findings and clarify how migration-related circumstances intersect with socioeconomic inequalities to shape early childhood diets.

## 6. Conclusions

Migration-related characteristics were associated with differences in the consumption of several UPF items among young children in Portugal. These associations were only partially explained by parental education, underscoring the role of broader socioeconomic and structural conditions that are unevenly distributed across migrant and non-migrant families. Although causal pathways cannot be established, the findings highlight population groups that may be differentially exposed to UPF-rich food environments during early childhood. From a public health perspective, these patterns warrant attention, as early-life dietary exposures are socially patterned and may contribute to later health inequalities. Policies and interventions aimed at improving food environments in early childhood settings, particularly in socially and economically disadvantaged contexts, may help reduce disparities in UPF exposure across diverse family backgrounds.

## Figures and Tables

**Table 1:** Description of the dependent variables (ultra-processed foods and drinks).

	Dependent Variables (Full Name, as Appeared in the Survey Reported by the Parents)	Dependent Variables (Short Name)
1	Chocolate-flavored and packaged milk (with or without lactose) (such as Agros^®^, Ucal^®^)	Flavored milk
2	Yogurts with or without lactose, flavored, solid or liquid, with pieces, or combined with chocolate or cereals (such as Mix-In Yogurt with Smarties Kids^®^), and fermented milks (such as Actimel^®^)	Flavored yogurt
3	Plant-based drinks (soy, rice, almond, among others), plant-based alternatives to yogurts and fermented milks	Plant-based drinks
4	Soft drinks with and without gas (such as Bongo^®^, Fresky^®^), flavored waters (such as Luso^®^ Fruta), reconstituted powdered juices (such as Tang^®^)	Soft & sugary drinks
5	Nectars and concentrated juices made from fruits or vegetables (Compal^®^ type)	Industrial juices
6	Iced tea or herbal teas (like Pleno^®^)	Iced teas
7	Sliced bread, hamburger or hot dog buns, wraps, toast, bagels, breadsticks	Industrial breads
8	Sausages, hamburgers, nuggets, fish fingers, seafood delicacies/crab delights, pâtés	Sausages, hamburger, etc.
9	Plant-based meat substitutes (vegetarian burgers, veggie sausages, seitan, vegetarian pâté, among others)	Plant-based meat substitutes
10	Ham, salami, mortadella, paio sausage, chorizo	Ham, salami, etc.
11	Margarine, chocolate/cocoa and hazelnut spreads (like Nutella^®^, Tulicreme^®^)	Margarine or other spreads
12	Spreadable/melted cheese (like The Laughing Cow^®^), petit suisse (like Philadelphia^®^, Danonino^®^)	Industrial cheese
13	Frozen french fries or those from fast-food restaurants (like McDonald’s) and frozen and instant mashed potatoes	Frozen or fast-food fries
14	Sauces (any type, including salad dressings), mustard, ketchup, and mayonnaise	Industrial sauces
15	Ready-made foods (hamburgers, fast food, salads with dressings)	Ready-to-eat food
16	Pizza, frozen or from fast-food restaurants like Pizza Hut^®^ or Telepizza^®^	Industrial or fast-food pizza
17	Pre-cooked foods (frozen and canned/packaged meals, pasta)	Precooked food
18	Bread snacks (such as Crackers, Gran Pavesi^®^, Tuc^®^), savory snacks, salted/sweet popcorn and packaged fried snacks (such as Cheetos^®^, Pringles^®^, Doritos^®^)	Bread-based snacks & chips
19	Commercial cookies and biscuits with or without filling (such as Oreo^®^, Chips Ahoy^®^), cereal bars (such as Chocapic^®^, Fitness^®^)	Cookies
20	Sugary cereals (such as Fitness^®^, Estrelitas^®^, Chocapic^®^, Golden Grahams^®^, Lion^®^)	Sugary cereals
21	Cereal flour, either dairy-based or non-dairy-based (such as Nestum^®^ or Cerelac^®^)	Dairy/non-dairy cereal flour
22	Cakes, pies, croissants, pancakes, crepes and other baked goods with or without cream packaging (such as Chipicao^®^, Bollycao^®^, Manhãzitos^®^)	Industrial cakes
23	Jams and fruit jellies	Fruit jellies and jams
24	Milk ice cream, cream ice cream and ice cream	Ice cream/sorbets
25	Puddings and dairy desserts (such as chocolate mousse, cream, vanilla cream, chocolate, caramel)	Puddings & dairy desserts
26	Gelatin (ready to eat or in powder form)	Gelatin
27	Chocolates and chocolate snacks (like Kinder Bueno^®^, KitKat^®^), candies and chewing gum, caramels, chewing gum	Chocolate & candies

**Table 2:** Characteristics of the sample (*n* = 682); *n* varies due to missing data.

		*n* (%)
Sex	Girls	327 (47.9%)
	Boys	355 (52.1%)
Age, mean (SD)		5.6 (1.0)
Child has always lived in Portugal	No	55 (8.1%)
	Yes	622 (91.9%)
Parents nationality	Two foreign parents	54 (8.1%)
	One foreign parent	58 (8.7%)
	Two Portuguese parents	554 (83.2%)
Mother’s origin	Africa	21 (3.1%)
	South America	45 (6.7%)
	Europe	18 (2.7%)
	Portugal	588 (87.5%)
Father’s origin	Africa	22 (3.3%)
	South America	44 (6.6%)
	Europe	12 (1.8%)
	Portugal	584 (88.2%)
Mother’s education	Below university	236 (36.4%)
	University	412 (63.6%)
Father’s education	Below university	320 (51.5%)
	University	301 (48.5%)

**Table 3:** Association between having lived outside Portugal and children’s UPF consumption (ref. category is “has always lived in Portugal”).

Dependent Variables Yesterday the Child Consumed…		Independent Variable (Ref. Always Lived in Portugal)	Model 1: OR (95% CI), *p* Crude	Model 2: OR (95% CI), *p* Adjusted for Sex, Age	Model 3: OR (95% CI), *p* Adjusted as M2 Plus SES
1	Flavored milk	No	2.06 (1.10; 3.85), 0.03 *	1.97 (1.05; 3.70), 0.04 *	1.94 (0.93; 4.02), 0.08 *
3	Plant-based drinks	No	3.01 (1.37; 6.61), 0.01 **	3.12 (1.41; 6.90), 0.01 **	1.54 (0.52; 4.61), 0.44
5	Industrial juices	No	3.24 (1.80; 5.85), <0.001 ***	3.19 (1.76; 5.76), <0.001 ***	2.66 (1.38; 5.11), 0.00 **
7	Industrial breads	No	4.14 (2.32; 7.39), <0.001 ***	4.10 (2.29; 7.33), <0.001 ***	5.34 (2.86; 9.99), <0.001 ***
9	Plant-based meat substitutes	No	7.68 (2.17; 27.23), 0.00 **	7.01 (1.96; 25.15), 0.00 **	15.29 (3.06; 76.35), 0.00 **
11	Margarine or other spreads	No	2.28 (1.21; 4.28), 0.01 *	2.22 (1.18; 4.20), 0.01 *	1.61 (0.77; 3.36), 0.20
13	Frozen or fast-food fries	No	2.83 (1.33; 5.99), 0.01 **	2.90 (1.36; 6.18), 0.01 **	2.15 (0.89; 5.21), 0.09
14	Industrial sauces	No	4.01 (1.96; 8.20), <0.001 ***	4.12 (2.00; 8.50), <0.001 ***	3.43 (1.48; 7.95), 0.00 **
19	Cookies	No	1.95 (1.10; 3.45), 0.02 *	1.93 (1.09; 3.43), 0.03 *	1.85 (0.99; 3.46), 0.05
22	Industrial cakes	No	2.58 (1.38; 4.80), 0.00 **	2.55 (1.36; 4.76), 0.00 **	2.10 (1.04; 4.22), 0.04 *

Reference category: Child did not consume the item on the previous day. Items with *p* > 0.05 in Model 1 were not considered, including: 2. Flavored yogurt, 4. Soft & sugary drinks, 6. Iced teas, 8. Sausages, hamburger, etc., 10. Ham, salami, etc., 12. Industrial cheese, 15. Ready-to-eat food, 16. Industrial or fast-food pizza, 17. Precooked food, 18. Bread-based snacks & chips, 20. Sugary cereals, 21. Dairy/non-dairy cereal flour, 23. Fruit jellies and jams, 24. Ice cream/sorbets, 25. Puddings & dairy desserts, 26. Gelatin, 27. Chocolate & candies. OR: odds ratio. CI: confidence interval. * *p* < 0.05, ** *p* < 0.01, *** *p* < 0.001.

**Table 4:** Association between parental nationality and children’s UPF consumption (ref. category is two Portuguese parents).

Dependent Variables Yesterday the Child Consumed…		Independent Variable (Ref. 2 Portuguese Parents)	Model 1: OR (95% CI), *p* Crude	Model 2: OR (95% CI), *p* Adjusted for Sex, Age	Model 3: OR (95% CI), *p* Adjusted as M2 Plus SES
1	Flavored milk	2 foreign parents	3.54 (1.91; 6.57), <0.001 ***	3.53 (1.89; 6.57), <0.001 ***	2.73 (1.31; 5.71), 0.01 **
		1 foreign parent	1.48 (0.76; 2.86), 0.25	1.48 (0.76; 2.88), 0.24	1.25 (0.54; 2.87), 0.60
3	Plant-based drinks	2 foreign parents	5.01 (2.33; 10.78), <0.001 ***	5.00 (2.32; 10.80), <0.001 ***	3.23 (1.22; 8.51), 0.02 *
		1 foreign parent	2.01 (0.80; 5.05), 0.14	1.95 (0.77; 4.92), 0.16	1.61 (0.53; 4.83), 0.40
4	Soft & sugary drinks	2 foreign parents	2.78 (1.39; 5.54), 0.00 **	2.78 (1.39; 5.54), 0.00 **	1.42 (0.59; 3.40), 0.43
		1 foreign parent	1.76 (0.87; 3.58), 0.12	1.77 (0.87; 3.60), 0.12	1.85 (0.78; 4.39), 0.16
5	Industrial juices	2 foreign parents	5.50 (2.99; 10.11), <0.001 ***	5.49 (2.98; 10.12), <0.001 ***	4.46 (2.26; 8.78), <0.001 ***
		1 foreign parent	1.80 (0.97; 3.35), 0.06	1.79 (0.96; 3.34), 0.07	1.58 (0.77; 3.22), 0.21
7	Industrial breads	2 foreign parents	6.02 (3.32; 10.92), <0.001 ***	6.01 (3.31; 10.90), <0.001 ***	8.09 (4.10; 15.98), <0.001 ***
		1 foreign parent	1.52 (0.80; 2.89), 0.20	1.52 (0.80; 2.89), 0.20	1.36 (0.65; 2.86), 0.42
9	Plant-based meat substitutes	2 foreign parents	6.16 (1.49; 25.47), 0.01 *	5.98 (1.44; 24.93), 0.01 *	11.98 (2.12; 67.65), 0.01 **
		1 foreign parent	3.41 (0.67; 17.31), 0.14	3.44 (0.67; 17.57), 0.14	4.41 (0.42; 46.77), 0.22
11	Margarine or other spreads	2 foreign parents	3.59 (1.96; 6.57), <0.001 ***	3.58 (1.95; 6.57), <0.001 ***	2.54 (1.25; 5.14), 0.01 *
		1 foreign parent	1.10 (0.54; 2.27), 0.79	1.09 (0.53; 2.25), 0.81	1.12 (0.50; 2.50), 0.79
12	Industrial cheese	2 foreign parents	1.28 (0.60; 2.74), 0.53	1.28 (0.60; 2.73), 0.53	1.03 (0.41; 2.57), 0.96
		1 foreign parent	1.94 (1.01; 3.72), 0.04 *	1.94 (1.01; 3.73), 0.04 *	2.78 (1.38; 5.61), 0.00 **
13	Frozen or fast-food fries	2 foreign parents	4.12 (1.99; 8.52), <0.001 ***	4.14 (2.00; 8.57), <0.001 ***	3.91 (1.72; 8.87), 0.00 **
		1 foreign parent	2.50 (1.14; 5.47), 0.02 *	2.44 (1.11; 5.35), 0.03 *	2.35 (0.91; 6.06), 0.08
14	Industrial sauces	2 foreign parents	4.73 (2.32; 9.65), <0.001 ***	4.78 (2.33; 9.78), <0.001 ***	3.98 (1.73; 9.16), 0.00 **
		1 foreign parent	1.27 (0.48; 3.37), 0.63	1.23 (0.46; 3.25), 0.68	1.05 (0.30; 3.67), 0.94
15	Ready-to-eat food	2 foreign parents	3.17 (1.01; 9.95), 0.04 *	3.18 (1.01; 9.99), 0.04 *	1.83 (0.38; 8.92), 0.46
		1 foreign parent	2.06 (0.58; 7.34), 0.27	2.03 (0.57; 7.24), 0.28	1.19 (0.15; 9.59), 0.87
18	Bread-based snacks & chips	2 foreign parents	2.27 (1.21; 4.29), 0.01 *	2.27 (1.20; 4.28), 0.01 *	1.66 (0.78; 3.53), 0.19
		1 foreign parent	1.32 (0.67; 2.59), 0.42	1.33 (0.68; 2.63), 0.41	1.50 (0.71; 3.19), 0.29
19	Cookies	2 foreign parents	2.30 (1.27; 4.17), 0.01 *	2.29 (1.26; 4.15), 0.01 *	2.23 (1.14; 4.37), 0.02 *
		1 foreign parent	0.78 (0.44; 1.39), 0.40	0.78 (0.44; 1.38), 0.39	0.95 (0.51; 1.78), 0.88
22	Industrial cakes	2 foreign parents	2.06 (1.08; 3.94), 0.03 *	2.07 (1.08; 3.95), 0.03 *	2.00 (0.96; 4.15), 0.06
		1 foreign parent	0.66 (0.29; 1.49), 0.31	0.64 (0.28; 1.47), 0.30	0.49 (0.17; 1.40), 0.18
23	Fruit jellies and jams	2 foreign parents	1.09 (0.38; 3.19), 0.87	1.09 (0.38; 3.19), 0.87	1.66 (0.55; 5.01), 0.37
		1 foreign parent	2.35 (1.08; 5.14), 0.03 *	2.36 (1.08; 5.16), 0.03 *	2.50 (1.07; 5.81), 0.03 *
26	Gelatin	2 foreign parents	2.56 (1.20; 5.44), 0.02 *	2.57 (1.21; 5.47), 0.01 *	2.18 (0.95; 5.00), 0.07
		1 foreign parent	0.97 (0.37; 2.55), 0.96	0.96 (0.37; 2.52), 0.94	0.78 (0.23; 2.66), 0.69

Reference category: Child does did not eat yesterday. Items with *p* > 0.05 in Model 1 were not considered, including: 2. Flavored yogurt, 6. Iced teas, 8. Sausages, hamburger, etc., 10. Ham, salami, etc., 16. Industrial or fast-food pizza, 17. Precooked food, 20. Sugary cereals, 21. Dairy/non-dairy cereal flour, 24. Ice cream/sorbets, 25. Puddings & dairy desserts, 27. Chocolate & candies. OR: odds ratio. CI: confidence interval. * *p* < 0.05, ** *p* < 0.01, *** *p* < 0.001.

## Data Availability

There are restrictions on the availability of data for this trial due to the signed consent agreements around data sharing, which only allow access to external researchers for studies following the project purposes. Requestors wishing to access the trial data used in this study can make a request to drdc@uc.pt.
